# 

**Published:** 1858

**Authors:** 


### *Receipts on Account of Medical Journal, by J. C. Hughes.*

| | | | |
|---|---|---|---|
| Dr. Armstong J. W. | $2 00 | Dr. Collings J. S. | $2 00 |
| Egleston J. Q. | 2 00 | Ewing G. | 2 00 |
| Floyd Maj. | 2 00 | Gillet S. G. | 2 00 |
| Lapslee S. M. | 2 00 | Moor Robt. | 2 00 |
| McKee Sam'l. | 2 00 | Newell O. W. | 2 00 |
| Ross " | 2 00 | Rillman J. R. | 2 00 |
| Rodgers " | 1 00 | Udell & Sawyer, | 2 00 |

### Books Received.

We take pleasure in acknowledging the receipt of a number of valuable Medical books from the publishing house of Blanchard & Lee, Philadelphia.

"ELEMENTS OF PATHOLOGICAL ANATOMY," by Samuel D. Gross, M. D. Third Edition, modified and thoroughly revised; illustrated by three hundred and forty engravings on wood; dedicated to Daniel Drake, M D., who was one of the earliest friends and patrons of Pathological Anatomy in the United States.

The name of the author is a sufficient guarantee for this work, and it will, no doubt, find a place in most of the medical libraries of the country. We are only sorry to see that the colored plates which held a place in the first edition have been left out in the present issue.

TODD & BOMAN'S "PHYSIOLOGICAL ANATOMY AND PHYSIOLOGY OF MAN," complete in one volume, with two hundred and ninety-eight illustrations.

This is a work of nearly one thousand pages, and well worth perusal.

LUDLOW'S "MANUAL OF MEDICAL EXAMINATIONS," especially designed for medical students.

This is a work of nearly eight hundred pages, containing a little of every thing relating to medicine—valuable as a book of reference preparatory to Examination but not containing enough to be of much value to the practitioner.

"WILSON ON DISEASES OF THE SKIN," fourth American, from the fourth and enlarged London edition.

This is a valuable work and should be in the hands of every physician. The same author publishes a series of plates illustrating the present work, but which has not accompanied this, and are sold separate.

"PRIZE ESSAYS ON CONSUMPTION." 1st. The effects of climate on Tuberculous Disease, by Edwin Lee, M. R. C. S., London. Being the dissertation to which the Fiske fund prize was awarded, June 6th, 1855.

2nd. The influence of Pregnancy on the development of Tubercles, by Edwin Warren, M. D., of Edenton, N. C. Being the dissertation to which the Fiske fund prize was awarded, June 4th, 1856.

These no doubt contain many valuable suggestions, as we have not had time to peruse them cannot say more.

"MILLER'S PRACTICE OF SURGERY." Revised by the American editor. Fourth American, from the last Edinburg edition. Illustrated by three hundred and sixty-four engravings on wood.

This work is valuable, and when accompanied with the "Principles," by the same author, one among our most valuable books of reference in Surgery.

"EVE'S SURGICAL CASES," dedicated to Charles D. Meigs, M. D.

This is a collection of interesting cases in Surgery by the author. The cases seem to have been selected with great care, making it a valuable work for reference.

